# Retrospective analysis of Schlafen11 (SLFN11) to predict the outcomes to therapies affecting the DNA damage response

**DOI:** 10.1038/s41416-021-01560-1

**Published:** 2021-10-18

**Authors:** Sophie E. Willis, Claudia Winkler, Martine P. Roudier, Tarrion Baird, Paola Marco-Casanova, Emma V. Jones, Philip Rowe, Jaime Rodriguez-Canales, Helen K. Angell, Felicia S. L. Ng, Paul M. Waring, Darren Hodgson, Jonathan A. Ledermann, Johanne I. Weberpals, Emma Dean, Elizabeth A. Harrington, J. Carl Barrett, Andrew J. Pierce, Elisabetta Leo, Gemma N. Jones

**Affiliations:** 1 Translational Medicine, Oncology R&D, AstraZeneca, Cambridge, UK; 2 Bioscience, Oncology R&D, AstraZeneca, Cambridge, UK; 3 GMD, Oncology R&D, AstraZeneca, Macclesfield, UK; 4 Translational Medicine, Oncology R&D, AstraZeneca, Gaithersburg, MD USA; 5 Translational Medicine, Oncology R&D, AstraZeneca, Boston, MA USA; 6 Cancer Research UK and UCL Cancer Trials Centre, UCL Cancer Institute, London, UK; 7 Ottawa Hospital Research Institute, Ottawa, ON Canada; 8 Clinical, Oncology R&D, AstraZeneca, Cambridge, UK

**Keywords:** Tumour biomarkers, Tumour biomarkers

## Abstract

**Background:**

The absence of the putative DNA/RNA helicase Schlafen11 (SLFN11) is thought to cause resistance to DNA-damaging agents (DDAs) and PARP inhibitors.

**Methods:**

We developed and validated a clinically applicable SLFN11 immunohistochemistry assay and retrospectively correlated SLFN11 tumour levels to patient outcome to the standard of care therapies and olaparib maintenance.

**Results:**

High SLFN11 associated with improved prognosis to the first-line treatment with DDAs platinum-plus-etoposide in SCLC patients, but was not strongly linked to paclitaxel–platinum response in ovarian cancer patients. Multivariate analysis of patients with relapsed platinum-sensitive ovarian cancer from the randomised, placebo-controlled Phase II olaparib maintenance Study19 showed SLFN11 tumour levels associated with sensitivity to olaparib. Study19 patients with high SLFN11 had a lower progression-free survival (PFS) hazard ratio compared to patients with low SLFN11, although both groups had the benefit of olaparib over placebo. Whilst caveated by small sample size, this trend was maintained for PFS, but not overall survival, when adjusting for BRCA status across the olaparib and placebo treatment groups, a key driver of PARP inhibitor sensitivity.

**Conclusion:**

We provide clinical evidence supporting the role of SLFN11 as a DDA therapy selection biomarker in SCLC and highlight the need for further clinical investigation into SLFN11 as a PARP inhibitor predictive biomarker.

## Background

The absence of Schlafen11 (SLFN11) has been linked to resistance to a wide range of DNA-damaging agents (DDA) such as fluoroindenoisoquinolines, nanoliposomal irinotecan, trabectidin, platinum drugs as well as PARP inhibitors (PARPi) [1–8]. The resistance of SLFN11 deficient cells to DDAs has been linked to the role of SLFN11 in the intra-S-phase checkpoint, in response to replication stress induced by DDAs and independent of Ataxia-telangiectasia-mutated and Rad3-related (ATR) [9]. SLFN11 causes early S-phase arrest and cell death in response to DNA damage, whereas cells deficient in SLFN11 were observed to slowly progress through to G2-phase and have a survival advantage. Specifically, SLFN11 is thought to block replication by changing replication fork chromatin structure subsequent to the ATR-mediated replication stress response. This interaction is thought to stabilise stalled replication forks during the intra-S and G2/M DNA damage checkpoints and suppress additional replication origin firing [10].

There is strong pre-clinical evidence that the presence of SLFN11 protein is associated with higher sensitivity to DDA treatment, but not non-DDA, but direct clinical investigation of SLFN11 as a DDA patient stratification biomarker is limited [11]. Recent reports have linked high levels of SLFN11 expression to sensitivity to platinum-based chemotherapy in gastric cancers [8] and to sensitivity to nedaplatin chemoradiotherapy in oesophageal squamous cell carcinoma patients [12]. Others have shown the absence of SLFN11 expression due to CpG promoter island hypermethylation in ovarian cancers linked to reduced OS in patients treated with cisplatin and carboplatin [4]. Also, high SLFN11 mRNA levels were associated with longer metastasis-free survival and overall survival (OS) in TNBC patients receiving anthracycline-based chemotherapy [13].

Several reports support a link between SLFN11 low/negative expression and resistance to PARP inhibitors [9, 14–16]. However, this has not been consistently observed for olaparib (Lynparza), the first-in-class PARP inhibitor. Recent research from our group has found knock-out of SLFN11 in a prostate cell line did not cause resistance to olaparib nor did low SLFN11 levels in triple-negative breast cancer (TNBC)-rich cohort of PDX models [11]. Whilst in SCLC cells high levels of SLFN11 were associated with PARPi sensitivity, this association was seen to a greater extent with the very potent PARP trapper talazoparib [14]. In addition, a Phase II trial identified patients with SLFN11-negative SCLC as those with worse progression-free survival (PFS) and OS to temozolomide in combination with the PARPi veliparib, compared with temozolomide with placebo [17]. Further investigation is needed, particularly in ovarian and breast cancers, to determine whether SLFN11 levels play a role in the response to olaparib in the clinic, and whether this is independent of BRCA mutations [18].

## Methods

### SLFN11 immunohistochemistry (IHC)

Three anti-SLFN11 antibodies (Supplementary Table 1) were screened at a range of concentrations and pH 6 and pH 9 retrievals. Final SLFN11 IHC protocol was performed on Leica BondRX; dewax, ER1(pH 6) retrieval 100 °C 25 min, SignalStain^®^ diluent (CST) block 10 min, F standard protocol (Leica, Bond polymer-refine-detection), without post-primary, DAB enhancer. SLFN11 antibody ab121731 (Abcam), diluted in Dako antibody diluent with background reducing components was applied for 15 min at 2.5 µg/ml in human tissues or 0.5 µg/ml in xenografts. Negative diluent only or matched concentration rabbit IgG (Abcam) controls were used.

### IHC pathology scoring

IHC slides were scanned at x20 on Aperio AT2 scanner (Leica). SLFN11 stained human tissues were evaluated by a pathologist for H-score, percentage positive SLFN11 and sub-clonality. H-score is calculated by estimating the proportion of stained tumour cells and the intensity of cell staining (grouped as 0, 1 + , 2+ or 3+ for negative, weak, intermediate and strong staining respectively) and applying the calculation H-score = (%1 + x 1)+(%2 + x 2)+(%3 + x 3) to produce a value between 0 and 300, where 300 is equal to 100% of cells with 3+ staining. Samples were excluded if internal control staining of stromal/endothelial cells was below IHC intensity 2 + . The pathologist was blinded to clinical outcomes during the scoring process.

### Ki67 immunohistochemistry (IHC)

Ki67 IHC was performed on the Labvision autostainer (ThermoFisher), deparaffinization in xylene and alcohol, pH 6 antigen retrieval 110 °C 5 min, 3% hydrogen peroxide and serum-free protein block (Dako). Primary antibody anti-Ki67 (Dako M7240) was diluted 1:100 in Dako antibody diluent. Detection used Dako envision + /HRP and DAB + (Dako). Carazzi’s haematoxylin counterstain. Percentage positive cells were quantified in the tumour regions by a pathologist.

### Animal studies

Xenografts were generated by the growth of DU145 or HT29 cell lines in nude mouse models. All studies were run in the UK and in accordance with AstraZeneca Global Bioethics Policy, UK Home Office legislation and Animal Scientific Procedures Act 1986.

### Human tissues

SLFN11 expression was evaluated using multi-normal (Supplementary Table 2), multi-tumour and colorectal tumour-specific (Supplementary Table 3) tissue microarrays (TMAs) obtained from Tristar. A set of breast cancer (*n* = 7) and platinum-resistant high-grade serous ovarian carcinoma (*n* = 7) resection specimens were obtained from Asterand.

A cohort of SCLC patients, selected on the availability of surgically resected FFPE material and clinical follow-up data were obtained, with appropriate patient consent and ethical approval, from Tristar, Avaden Biosciences, Nottingham hospital, Asterand, Cureline, TransHit, Proteogenix. Supplementary Tables 4–8 list the vendor-provided demographic details of 124 SCLC patients and 110 high-grade serous ovarian cancer (HGSOC) patients from Study19 (NCT00753545) randomised Phase II olaparib trial. All Study19 samples were presented in a tissue microarray (TMA) format [19]. Samples from 34 HGSOC patients with extremely good (*n* = 17) or poor (*n* = 17) responses to first-line carboplatin-plus-paclitaxel were provided by Dr. Johanne Weberpals [20]. All human tissue samples were obtained under AstraZeneca’s global bioethics policy (https://www.astrazeneca.com/content/dam/az/our-company/Documents/Bioethics-Policy.pdf).

### Cell culture

DU145 cells (DSMZ) and DU145 CRISPR-Cas-9 SLFN11 knock-out cell line generated by AstraZeneca (Oncology Bioscience UK) cultured in EMEM media with 10% foetal bovine serum (ATCC). Identities were verified by short tandem repeat analysis and mycoplasma screened. Cells were processed into formalin-fixed paraffin-embedded (FFPE) blocks as described in [21].

### Western blot

Cells were lysed in RIPA lysis buffer (ThermoFisher) with phosphatase and protease inhibitors (Sigma). Protein quantification by BCA assay (ThermoFisher). Protein electrophoresis on 4–12% Bis-Tris MidiGel (LifeTech), transferred to a nitrocellulose membrane (iBLOT), blocked in 5% milk in TBS-T (0.05%) and probed for anti-SLFN11 (ab121731, Abcam, 1:1000 dilution) and GAPDH loading control (14C10, CST, 1:3000 dilution). Detection by HRP-linked goat anti-rabbit secondary antibodies, SuperSignal WestDura ECL detection reagents (ThermoFisher) and standard film.

### RNA in situ hybridisation

In situ detection of *SLFN11* mRNA transcripts was performed using the RNAScope LS Red Kit (Advanced Cell Diagnostics) according to the manufacturer’s protocol [22]. Signal visualised using Bond Polymer Refine Red Kit (Leica). Positive (*PPIB)* and negative (*DapB)* control probes were used.

### Mass spectrometry

Mass spectrometry carried out at Oncoplex using 10-µm FFPE sections. Tumour extracted using laser capture microdissection, trypsin digested and unique control isotopically labelled tryptic peptides for SLFN11 were added. Samples were run on a triple quadrupole LC/MS/MS, and the ratio of patient endogenous peptide to control isotopically labelled peptide provided quantitative protein concentration. An SLFN11-positive sample was defined as any sample with a value above the limit of quantification.

### NanoString gene expression

5–10-µm FFPE sections were used for NanoString gene expression (GE) analysis. Tumour macro-dissected and RNA extracted with RNeasy FFPE extraction kit (Qiagen). NanoString carried out following the manufacturer’s instructions (fixed 21-h hybridisation time, 100 ng input RNA) and the AZ-designed DDRmax code set, bespoke 800 gene code set covering DNA Damage Response gene pathways. SLFN11 probe design is given in Supplementary Fig. 1. Cartridges read on the  nCounter GEN2 Digital Analyzer station with high-resolution selected (3 h enhanced, 555 fields of view captured). Log2 normalised data exported for analysis after internal positive control, and housekeeping gene normalisation using nSolver Analysis Software version 4.0.

### Statistical analysis

Statistical significance achieved if *P* ≤ 0.05. Where two groups were analysed, the Mann–Whitney test was used and where more than two group analysed, the Kruskal–Wallis with Dunn’s multiple-comparisons was used. A paired *t* test was used to assess intra-tumour differences between subclones. Linear regression was used for correlation analysis and a log-rank (Mantel–Cox) test was used for survival analysis.

To select H-score cut-offs, *P* values were generated for OS difference between high and low SLFN11 using different SLFN11 H-score cut-offs. This was carried out using a linear regression-based model with a dichotomous grouping variable, where dichotomy has been conducted at different SLFN11 H-score cut-off points and H-score was replaced by the binary categorical variable. The H-score where the *P* value was lowest was selected as the cut-off.

An adjusted Cox proportional hazards model used to assess PFS and OS differences in Study19, using methods and caveats previously described [23]. Multivariate analysis adjusted for patient BRCA status carried out using same Cox proportional hazards model, adjusted for BRCA status.

## Results

### Validation of a clinically applicable SLFN11 IHC assay

We developed a spatially resolved immunohistochemistry (IHC) assay to detect SLFN11 protein in clinical tissues. We followed stringent assay validation guidelines [24] and used appropriate controls to screen commercially available SLFN11 antibodies by IHC (Supplementary Table 1). Polyclonal ab121731 (Abcam) SLFN11 antibody was selected based on optimal specific signal to background staining. The selected antibody showed good nuclear specificity, demonstrated by complete loss of SLFN11 in DU145 CRISPR Cas-9 SLFN11 knock-out cells by IHC (Supplementary Fig. 2A) and western blot (Supplementary Fig. 2B). SLFN11 staining in DU145 and HT29 xenograft tissues were positive and negative respectively, consistent with gene and protein expression data [7] (Supplementary Fig. 2C).

**Fig. 1:**
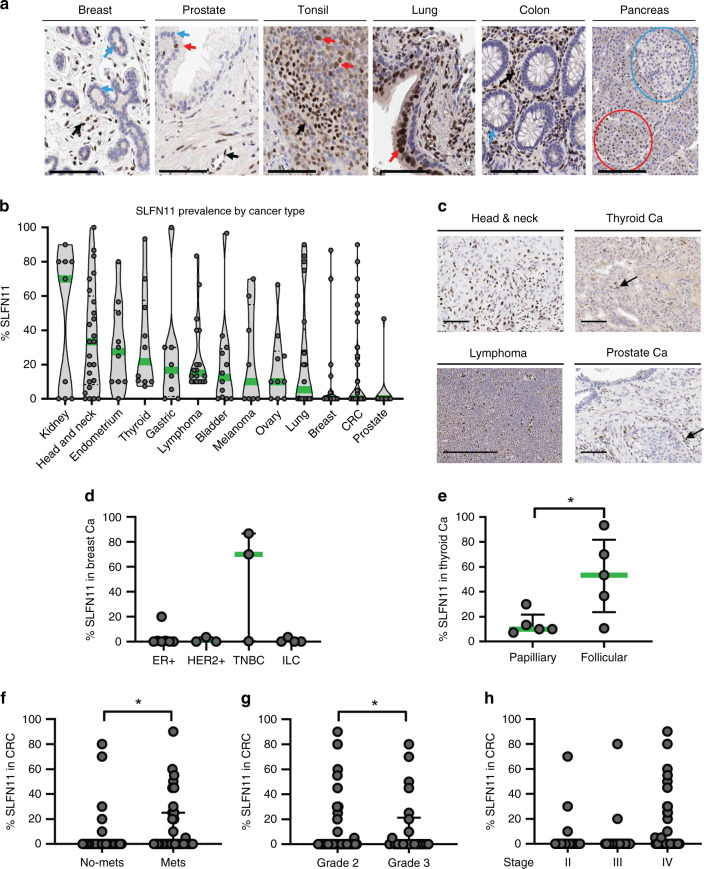
SLFN11 protein levels are variable across tumour types and higher levels are found in more aggressive cancer subtypes. **a** SLFN11 expression in normal tissues, arrows indicate (from left to right); negative breast lobular cells, positive (red) and negative (blue) prostatic cells, positive lymphocytes, positive bronchial epithelial cells, negative colon epithelial cells. Negative Langerhans islets (blue circle) and positive cluster of acinar cells (red circle) also shown. **b** SLFN11 prevalence in various cancer types from multi-tumour tissue microarrays (TMAs), expression of SLFN11 shown as percent of positive tumour cells when internal control staining was acceptable. Median given (green) for each cancer type. **c** Example images of SLFN11 IHC staining in head and neck, thyroid, lymphoma and prostate cancers (Ca). **d**, **e** Breakdown of the SLFN11 protein levels by: **d** breast cancer subtype; oestrogen receptor-positive (ER + ), human epidermal growth factor receptor 2 (HER2 + ), triple-negative breast cancer (TNBC), invasive lobular breast cancer (ILC) and **e** thyroid cancer subtype; papillary and follicular. **f**–**h** SLFN11 expression in colorectal cancer (CRC) patients (*n* = 144) subcategorised by clinical patient information: **f** presence of metastasis or not; **g** tumour grade, **h** disease stage. Data shown as median ± interquartile range. *P value <0.05 by Mann–Whitney test. Black arrows are stromal and endothelial cells. Scale bars at 100 µm.

In human tissues, ciliated bronchial cells, but not basal bronchial epithelium, and pancreatic acinar cells consistently expressed SLFN11, while most normal ductal or glandular epithelial cells in the breast, pancreas, prostate and colon were SLFN11 negative (Fig. 1a and Supplementary Table 2). SLFN11 positivity was consistently observed in stromal, endothelial and immune cells, independent of tumoural SLFN11 expression (Fig. 1a and Supplementary Fig. 2D), providing a valuable internal quality control against pre-analytical factors like inadequate tissue fixation. Standard isotype run controls ensured no unspecific staining from detection methods (Supplementary Fig. 2E).

### SLFN11-negative tumours found across tumour types

SLFN11 protein expression varied across 13 different tumour types assessed by multi-tumour TMAs; 37% of all tumours tested showed no SLFN11. The lowest median SLFN11 expressors were colon and prostate cancers, the highest were kidney and head and neck cancers (Fig. 1b, c), and these findings corroborated with prevalence studies in patient-derived-xenograft models [11] and clinical samples [12, 25].

SLFN11 was absent in tumour cells in 71% of patients with breast cancer (*n* = 17), the two highest SLFN11 expressors were TNBC (Fig. 1d). SLFN11 was higher in the rarer more aggressive follicular thyroid cancer (*n* = 5) compared to papillary subtype (*n* = 5) (*P* = 0.032) (Fig. 1e). No significant differences were found between different grades of lymphoma (*n* = 8 low grade; *n* = 10 high grade), histological subtypes of lung cancer (*n* = 9 adenocarcinoma, *n* = 7 squamous cell carcinoma, *n* = 3 SCLC) or histological subtypes of ovarian cancer (*n* = 6 serous, *n* = 2 mucinous, *n* = 2 endometroid). These data should be caveated by small numbers of samples for each tumour type and larger cohorts should be evaluated using this IHC assay to assess the association between SLFN11 levels and clinical outcomes in multiple cancer types.

Median SLFN11 levels in colorectal cancers (CRC) (*n* = 144) (Supplementary Table 3) were modestly higher in patients with metastatic cancer compared to locally advanced disease (*P* = 0.030, Fig. 1f), and in patients with higher tumoural grade 3 compared to grade 2 (*P* = 0.022, Fig. 1g), but did not differ by CRC stage (Fig. 1h). Whilst limitations include the use of a largely SLFN11-negative CRC population (80%) and no Stage I patients, these findings show SLFN11 positivity associates with poor prognosis clinical features in certain tumour types.

### SLFN11 sub-clonal expression

**Fig. 2:**
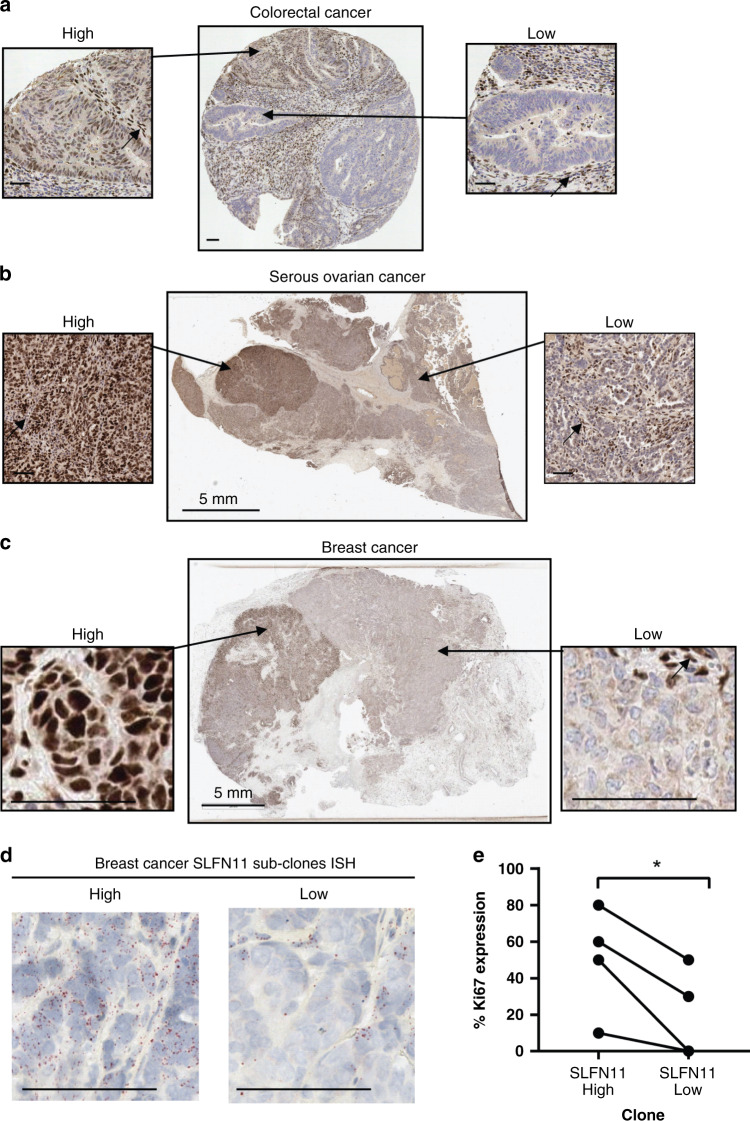
SLFN11 protein expression in clinical tissues can be sub-clonal, with both high- and low-expressing regions within a single tumour. **a**–**c** SLFN11 sub-clonal expression pattern demonstrated by IHC of a **a** CRC TMA core, **b** resected serous ovarian cancer tissue, **c** resected breast cancer tissue. A magnified image of the high- and low SLFN11 subclones are shown for each. Black arrows indicate stromal and endothelial cells used as a positive internal control. **d** RNA in situ hybridisation (ISH) of SLFN11 showing expression of SLFN11 RNA transcripts in the breast cancer SLFN11 subclones. **e** SLFN11 sub-clonal expression correlated to Ki67 expression. Line indicates patient-matched subclones. *P value < 0.05 paired *t* test. Scale bars at 50 µm unless otherwise stated.

In contrast to homogeneous cell/xenograft models, patient tumours consist of multiple sub-clonal cell populations that can differ in molecular profiles and evolve/expand in response to selective therapy or environmental pressures [26]. Detecting sub-clonal expression of patient stratification biomarkers could elucidate mechanisms of resistance and better inform therapy selections. IHC analysis identified a subset of tumours with sub-clonal SLFN11 expression, where spatially distinct high and low SLFN11 expressing subclones were identified within the same patient sample (Fig. 2a–c). Sub-clonality was rarely observed in TMAs (only *n* = 1 CRC TMA core) due to the small core size. In whole resections, sub-clonality was found in 6/34 HGSOC tumours (Fig. 2b) and 4/7 breast cancer tumours (Fig. 2c).

Subsequent in situ hybridisation spatial profiling of these subclones showed *SLFN11* RNA transcript levels paralleled SLFN11 protein expression patterns (Fig. 2d). In one case, histological features were also distinct between subclones with the high SLFN11 sub-clone presenting hyperchromatic-nuclei (Supplementary Fig. 3A), high SLFN11 subclones also had higher proliferative states, demonstrated by increased Ki67 (*P* = 0.035, Fig. 2e). Other methods of SLFN11 protein and RNA detection (mass spectrometry and NanoString gene expression), were not suitable for sub-clonality assessment and confounded by the inclusion of SLFN11-positive stromal and immune cells, resulting in discrepancies in concordance with SLFN11 IHC H-score (*R*^2^ = 0.82 and *R*^2^ = 0.70 respectively, Supplementary Fig. 3B, C).

### Low SLFN11 associated with poor prognosis in DDA-treated SCLC

**Fig. 3:**
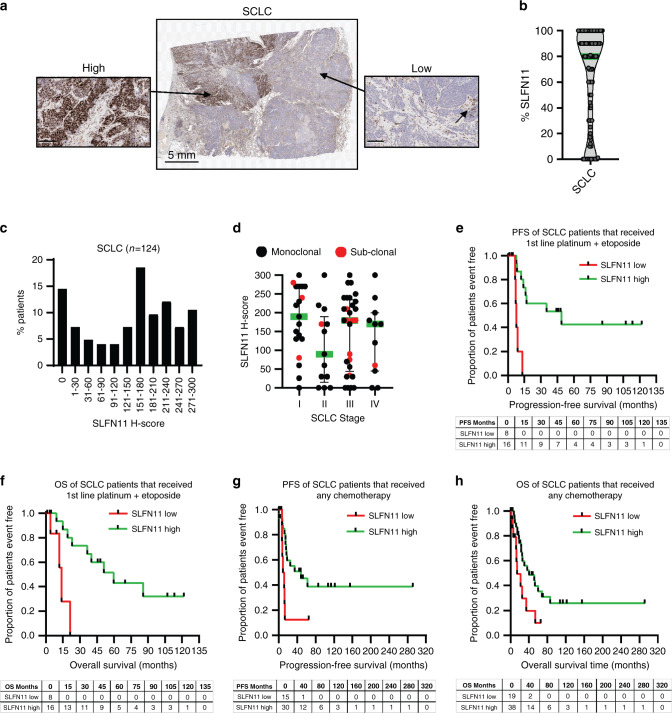
High SLFN11 in small-cell lung cancer (SCLC) patients treated with standard of care linked to improved clinical outcome. **a** Image of a SCLC patient tumour demonstrating sub-clonal SLFN11 expression, areas from the high- and low-expressing subclones are magnified. The black arrow indicates SLFN11-positive stromal cells in the negative sub-clone. Scale bars at 100 µm unless otherwise stated. **b** SLFN11 prevalence by IHC in SCLC patients, median in green. **c** Bimodal prevalence of SLFN11 in the SCLC cohort (*n* = 124). **d** SLFN11 expression in SCLC subcategorised by disease stage. Black; SLFN11 monoclonal expression, Red; SLFN11 sub-clonal tumours. Median ± interquartile range shown. **e**–**h** Kaplan–Meier analysis of SCLC patients categorised by SLFN11 expression; SLFN11 high (H-score >122) compared to low (H-score ≤122). **e** Progression-free survival (PFS) and **f** overall survival (OS) in months for patients (*n* = 24) that received first-line platinum (carboplatin or cisplatin) plus etoposide. **g** PFS and **h** OS in months for 45 and 57 patients, respectively, that received any chemotherapy. Events table with patients at risk shown for each timepoint.

In total, 15% of SCLC tumours presented with SLFN11 sub-clonality (*n* = 19/124) (Fig. 3a), and median positivity was generally high at 80% (Fig. 3b, c). An optimised H-score cut-off to divide SCLC patients into SLFN11 high- and low subgroups was identified as 122 using linear regression-based modelling of SLFN11 to OS in a randomly selected training subset of 38 SCLC samples. We confirmed there was no link between SLFN11 and disease stage, and sub-clonality was found across all stages (Fig. 3d and Supplementary Table 4).

SCLC standard of care DDAs includes carboplatin or cisplatin that induce bulky intrastrand DNA adducts, as well as the topoisomerase II poison etoposide [27]. We show SLFN11 high patients (>122 H-score) treated with first-line platinum (cisplatin or carboplatin) plus etoposide (*n* = 24) had significantly improved PFS (median 48.7 months) compared to SLFN11 low patients (median 7.8 months; *P* = 0.0002) (Fig. 3e). Similar results for this treatment group were found for OS, SLFN11 high patients living significantly longer (59.7 months) than low patients (13.9 months; *P* = 0.001) (Fig. 3f and Supplementary Table 5). High SLFN11 was also predictive of improved PFS and OS in 45 and 57 SCLC patients, respectively, with clinical follow-up, who had been treated with any chemotherapy; SLFN11 high median PFS 48.7 months and OS 40.2 months, SLFN11 low median PFS 10.3 months and OS 13.9 months (PFS *P* = 0.017 and OS *P* = 0.020) (Fig. 3g, h and Supplementary Tables 6 and 7). Sub-clonality did not associate with clinical outcome, Supplementary Fig. 4A, B.

### Uncertain role for SLFN11 in HGSOC paclitaxel–platinum sensitivity

**Fig. 4:**
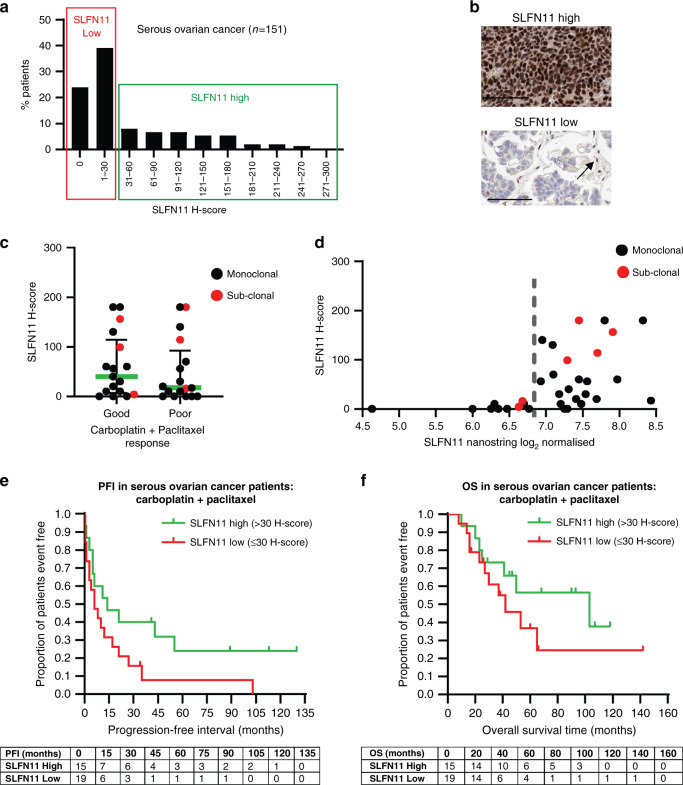
SLFN11 not significantly associated with sensitivity to the paclitaxel–carboplatin doublet in serous ovarian cancers. **a** Prevalence of SLFN11 by IHC H-score in serous ovarian cancer patient biopsies (*n* = 151), cut-off depicting the high and low SLFN11 subgroups shown, **b** representative images of high and low SLFN11 tumours. Black arrow indicates SLFN11-positive stromal cells used as an internal control. Scale bars at 100 µm. **c** SLFN11 H-score of patients divided by extreme good and poor clinical responders to carboplatin and paclitaxel doublet therapy. Median ± interquartile range shown. **d** SLFN11 IHC protein expression by H-score compared to NanoString quantified SLFN11 gene expression in the HGSOC patients. Dotted line indicates gene expression threshold that distinguishes positive from negative SLFN11 patients. Patients with sub-clonal SLFN11 indicated by a red dot. **e**, **f** Kaplan–Meier survival curves of (**e**) progression-free interval (PFI) and (**f**) overall survival (OS) of high (>30 H-score SLFN11) vs. low SLFN11 ( ≤ 30 H-score SLFN11) subgroups in the 34 patients with high-grade serous ovarian cancer treated with carboplatin and paclitaxel doublet. Events table with patients at risk shown for each timepoint.

SLFN11 expression was generally low in serous ovarian cancer samples, median H-score 20, 16% positivity (*n* = 151) (Fig. 4a, b and Supplementary Fig. 5A). The 122 H-score cut-off developed in SCLC was not appropriate for this low-expressing indication so instead, a 30 H-score cut-off was selected based on expression distribution (Fig. 4a).

DDAs such as carboplatin are standard of care in HGSOC, along with microtubule stabilising non-DDA paclitaxel that causes mitotic arrest [28]. We found no significant link between SLFN11 levels and sensitivity to first-line carboplatin-plus-paclitaxel chemotherapy in 34 HGSOC patients subgrouped by extreme good or poor responses (progression-free interval; PFI > 12 months or <6 months, respectively; *P* = 0.487 Fig. 4c) [20]. This was also true when SLFN11 was measured by gene expression (*P* = 0.432, Supplementary Fig. 5B), despite discordance between protein and gene expression techniques in this subset (*R*^2^ = 0.26; Fig. 4d), most likely confounded by the inclusion of stomal and immune cells. However, full survival data analysis identified longer median PFI in SLFN11 high patients of 14 months compared to 6 months in SLFN11 low patients, but differences did not reach statistical significance (*P* = 0.0705, Fig. 4e). The same trend was observed for OS with a median survival of 103 months in the SLFN11 high group compared to just 42 months in the low group (*P* = 0.2475, Fig. 4f). Sub-clonality did not associate with clinical outcome, but patient numbers were limited (Supplementary Fig. 5C, D). Further investigation is required to determine the role SLFN11 plays in paclitaxel–platinum sensitivity.

### High levels of SLFN11 associated with improved clinical outcome to olaparib in HGSOC

**Fig. 5:**
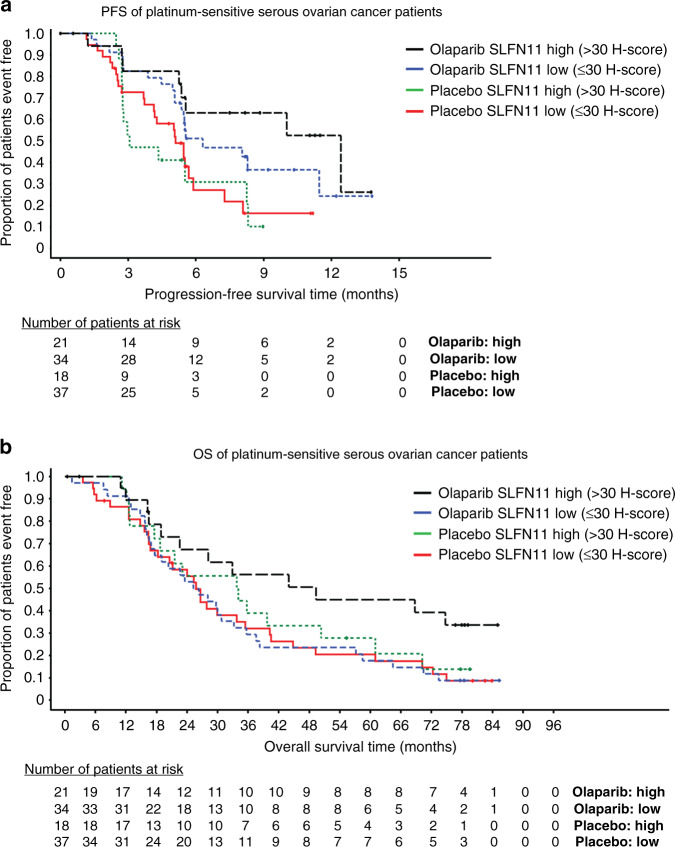
Elevated SLFN11 levels in HGSOC patients confers sensitivity to olaparib. **a** Cox proportional hazards model of progression-free survival (PFS) of high (>30 H-score SLFN11) vs. low SLFN11 ( ≤ 30 H-score SLFN11) subgroups in platinum-sensitive serous ovarian carcinoma patients treated with either placebo or olaparib. **b** Overall survival (OS) of high vs. low SLFN11 in the same study. Events table with patients at risk shown for each timepoint.

PARP inhibitors such as olaparib (Lynparza) are approved for use in the first-line maintenance setting in platinum-sensitive relapsed ovarian cancers by the FDA and EMA. We carried out retrospective exploratory analysis of 110 HGSOC patients from the Phase II randomised and placebo-controlled olaparib maintenance clinical trial (D0810C00019; NCT00753545), where samples were evaluable for SLFN11. We showed the PFS hazard ratio (HR) was numerically lower in the SLFN11 high group (>30 H-score), 0.28 HR [0.09, 0.74 95% CI], compared to the SLFN11 low group, HR 0.49 [0.26, 0.91 95% CI] (Fig. 5a), although both groups showed the benefit of olaparib over placebo. These findings are caveated by relatively wide confidence intervals due to sub-group size and a limited number of events, but clearly highlight a trend of high SLFN11 expression and better clinical outcome to olaparib. In support of this trend, SLFN11 high patients had a longer median PFS of 12.4 months in the olaparib arm compared to 3.1 months with placebo, whilst there was less difference in median PFS in the SLFN11 low group (6.3 months post olaparib and 5.1 months with placebo). A similar trend was observed with OS; SLFN11 high patients olaparib group had longer survival compared to placebo (median OS 49.3 months olaparib, 33.9 months placebo; HR 0.67 [0.30, 1.48 95% CI]), and compared to the SLFN11 low patients (median OS 25.8 months olaparib, 25.8 months placebo; HR 0.93 [0.56, 1.54 95% CI]) (Fig. 5b).

**Fig. 6:**
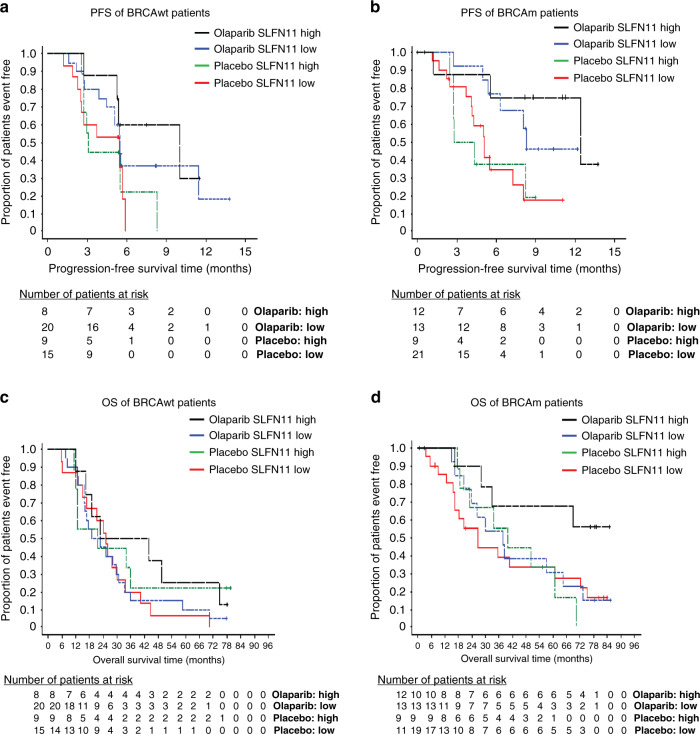
SLFN11 does not predict olaparib sensitivity independent of BRCA status. **a**–**d** Cox proportional hazards model by SLFN11 high (>30 H-score SLFN11) vs. low SLFN11 ( ≤ 30 H-score SLFN11) subgroups in platinum-sensitive serous ovarian carcinoma patients treated with either placebo or olaparib for (**a**) progression-free survival (PFS) in BRCA wild-type patients, **b** PFS in BRCA mutant patients, **c** Overall survival (OS) in BRCA wild-type patients, **d** OS in BRCA mutant patients. Events table with patients at risk shown for each timepoint. VUS BRCAm patients included in the BRCAwt group.

BRCA mutations are key drivers of PARP inhibitor sensitivity [19, 29], therefore to address potential confounding effects by BRCA mutation imbalances across subgroups (Supplementary Table 8 and Supplementary Fig. 6), multivariate analysis was subsequently carried out with adjustment for BRCA status. Results showed a similar trend as before, with improved PFS in the SLFN11 high group; HR 0.24 [0.07, 0.69 95% CI] compared to SLFN11 low; HR 0.38 [0.20, 0.72 95% CI], although again caveated by wide confidence intervals. In line with our previous observations, median PFS was longest in the SLFN11 high groups treated with olaparib (12.4 months BRCAm and 10 months BRCAwt), compared to the SLFN11 low groups (8.3 months BRCAm and 5.5 months BRCAwt), whilst median PFS in the placebo groups ranged from 3.1 to 5.5 months (Fig. 6a, b). However, there was no association between OS and SLFN11 positivity observed in multivariate analysis including BRCA status, with SLFN11 high HR 0.73 [0.31, 1.64 95% CI] vs. SLFN11 low HR 0.79 [0.47, 1.32 95% CI]. The OS Kaplan–Meier plots show OS benefit of olaparib over placebo in the BRCAm patients regardless of SLFN11 status and no clear difference in treatment effect on OS by SLFN11 status in the BRCAwt groups (Fig. 6c, d). These findings show that whilst SLFN11 is associated with sensitivity to olaparib in HGSOC, BRCA mutations are a stronger driver of PARP inhibitor sensitivity in this setting.

## Discussion

We developed a clinically applicable IHC assay to spatially assess SLFN11 expression patterns and prevalence across multiple tumour types and elucidated the role of SLFN11 as a potential biomarker of PARP inhibitor drug sensitivity. Our findings suggest high levels of SLFN11 may confer sensitivity to olaparib in platinum-sensitive ovarian cancer patients, but will not replace BRCAm as a key driver of sensitivity. Whilst in SCLC patients, high SLFN11 protein levels correlated to DDA sensitivity.

Our clinical data in SCLC confirms what others have consistently shown in pre-clinical models and a small number of clinical studies, that there is an association between low SLFN11 tumour expression and resistance to directly DNA-damaging therapies, particularly in this indication [3–7, 11, 17, 30, 31]. SLFN11 levels, whilst predictive of outcome to chemotherapy, did not significantly differ by disease stage in our SCLC or CRC cohorts, further highlighting SLFN11, not as a prognostic biomarker, but a predictive biomarker for DDA sensitivity. Although larger clinical studies are needed to confirm these preliminary observations that may be biased towards the resection sample type used.

High levels of SLFN11 in SCLC compared to other tumour types might be related to the high levels of intratumoural heterogeneity and transcriptional plasticity observed in this tumour type. Recent work suggests cisplatin resistance is related to the emergence of a sub-clone of cells that have switched transcriptional subtypes [32]. Further investigation into whether cisplatin-resistant subclones have lower levels of SLFN11 could be conducted using the spatially resolved IHC methods described here.

Evidence of SLFN11 as a patient stratification biomarker for DDA in clinical indications beyond SCLC is limited, although supportive clinical data were reported in oesophageal, gastric and TNBC [8, 12, 13]. In HGSOC, an indication known to have high BRCA mutation frequency (35%) [33], results from cohorts we analysed were less clear. Whilst others found high SLFN11 gene expression associates with better prognosis in cisplatin-treated ovarian cancer patients [5], we could not confidently determine if SLFN11 associated with improved prognosis to the standard of care carboplatin–paclitaxel doublet, but trends indicated longer PFI in SLFN11 high patients. The contribution of paclitaxel in this combination, which is not directly DNA damaging, may play a role in reducing the dependency of SLFN11 status on response. Experiments in pre-clinical models show SLFN11 silencing does not influence response to paclitaxel [7, 28], whilst SLFN11 plays a significant role in carboplatin sensitivity [7, 11]. It would be interesting to assess in a larger cohort, which would also allow the contribution of BRCA mutations to response to be assessed.

Clinical data consistently highlights the predominant role of BRCA mutations on PARP inhibitor sensitivity [19, 29, 34]. Recent studies suggesting a link between SLFN11 absence and PARP inhibitor resistance have attracted interest, but have typically been carried out in settings where BRCA mutations are rare, such as SCLC, and are complicated by the use of the PARP inhibitors in combination with DNA-damaging agents such as temozolomide [14, 15, 17, 35]. This study examined for the first time a randomised placebo-controlled Phase II maintenance trial of patients with HGSOC, to determine if SLFN11 is associated with clinical outcome to olaparib. Our findings demonstrate an interesting trend linking high SLFN11 expression with longer progression-free survival following olaparib that was independent of BRCA mutation, although sample sizes were limited and this finding did not extend to longer overall survival when BRCA status was accounted for. There are further biomarkers for PARPi response beyond BRCA mutation, including mutations in other homologous recombination repair (HRR) genes and gene signatures of homologous recombination deficiency, but the assessment of the relationship between SLFN11 levels and HRR mutations was not possible for this trial due to low frequency of non-BRCA HRR mutations. Other caveats include the use of archival samples from heavily pre-treated patients and tumours in a TMA format, preventing assessment of SLFN11 sub-clonality. We propose that high expression of SLFN11 may confer some benefit to olaparib in HGSOC, however, not to the same extent as BRCA mutations and loss of function HRR mutations that were previously analysed for this trial (NCT00753545) [19, 34].

There are ongoing studies investigating SLFN11 as a patient stratification biomarker for PARP inhibitors in SCLC [36, 37], but SLFN11 could also be used to inform on patients resistant to DDA that may benefit from combinations of DDA with DNA damage response inhibitors such as WEE1, CHK1 and ATR inhibitors [11, 38]. In support of this, recent work from our group in HGSOC patients demonstrated efficacy of the Wee1 inhibitor adavosertib in combination with gemcitabine, independent of tumour SLFN11 status (NCT02151292). However, data were underpowered to determine whether SLFN11 levels could predict response in the gemcitabine-placebo arm [39].

Finally, we highlight the importance of finding normal non-tumourigenic epithelial cells are mostly SLFN11-negative. This suggests SLFN11 expression may be increased during tumorigenesis, rather than ‘lost’, as it often referenced in publications. The notable exception observed was the lung, where SLFN11 levels were high in normal ciliated bronchial epithelia. however, this is unlikely to be reflected in bronchogenic squamous cell carcinoma which arises from metaplastic bronchial epithelial cells following carcinogen exposure [40–42]. Further investigation into SLFN11 changes during tumorigenesis and following therapeutic interventions would be of interest.

We strongly recommend future studies use a spatially resolving IHC method, which can also capture sub-clonal expression patterns of SLFN11, a potential method for early detection of negative sub-clonal populations that may cause therapeutic resistance [26]. We have shown differences in the prevalence of sub-clonality across different tumour types, but our findings have not demonstrated that the presence of sub-clonality is determinate of response and instead that overall SLFN11 levels are more predictive of response to DDAs. Larger studies are required to further investigate this, which do not use TMAs or small core-needle biopsies.

These findings highlight the complexities and need for context-dependent consideration of using SLFN11 to predict patient outcome to DDAs and DDR inhibitors in the clinical setting. We find elevated tumour SLFN11 not only linked to improved clinical outcome to chemotherapy in SCLC but to an extent is also associated with olaparib sensitivity in platinum-sensitive HGSOC patients. We have yet to fully understand the interplay between this novel cell cycle checkpoint and BRCA/HRR mutations, future work should build upon these initial findings to deepen our mechanistic understanding and inform of future use of SLFN11 as a patient selection biomarker.

## Supplementary information

Reproducibility Checklist

Retrospective analysis of Schlafen11 (SLFN11) to predict outcome to therapies affecting the DNA damage response Supplementary Material

## Data Availability

All the data supporting the findings of this study are available either within the supplementary information files or upon reasonable request from the corresponding authors.
