# Supplementary material for: Retrospective analysis of Schlafen11 (SLFN11) to predict the outcomes to therapies affecting the DNA damage response
Source: Br J Cancer. 2021 Oct 18;125(12):1666–76. doi: 10.1038/s41416-021-01560-1 (PMC8651811; doi:10.1038/s41416-021-01560-1)
Supplement: Supplementary file 2 — Retrospective analysis of Schlafen11 (SLFN11) to predict outcome to therapies affecting the DNA damage response Supplementary Material [file 41416_2021_1560_MOESM2_ESM.docx]

*Supplementary Table 1: Commercial SLFN11 antibodies available at the time of screening.*

Details of the three anti-SLFN11 antibodies that were screened for suitability in an IHC assay. Table provides the catalogue numbers, clone name where applicable, isotype details, recommended applications at the time of screening, antibody host, species reactivity, clonality and epitope if available.

| **Supplier** | **Catalogue Number** | **Clone** | **Isotope** | **Applications recommended by company** | **Host Source** | **Species Reactivity** | **Mono / Polyclonal** | **Epitope** |
| --- | --- | --- | --- | --- | --- | --- | --- | --- |
| **Merck** | MABF248 | 4G9 | IgM | WB, IP | Ms | Hu | Mono | Recombinant protein corresponding to human SLFN11 |
| **Abcam** | ab121731 | - | IgG | WB, ICC/IF, | Rb | Hu | Poly | Amino acids 255-333 of Human SLFN11 |
| **Novus** | NBP2-57084 | - | IgG | ICC/IF | Rb | Hu | Poly | ETSVRSMDSREAFCFLKTKRKPKILEEGPFHKIHKGVYQELPNSDPADPNSDPADLIFQKDYLEYGEILP |

*Supplementary Table 2: Number of normal tissue TMA cores evaluated by SLFN11 IHC and details of staining patterns observed.*

|  | Normal tissue cohort (epithelial cells) | | | |
| --- | --- | --- | --- | --- |
|  | SLFN11 negative | | SLFN11 positive | |
|  | n | % | n | % |
| Bladder urothelium | 5 | 100 | 0 | 0 |
| Kidney renal cortex | 7 | 100 | 0 | 0 |
| Colon | 7 | 100 | 0 | 0 |
| Bronchus^a^ | 4 | 100 | 0 | 0 |
| Alveolar Lung tissue^b^ | 2 | 100 | 0 | 0 |
| Lymph nodes^c^ | 0 | 0 | 7 | 100 |
| Pancreas^d^ | 5 | 56 | 4 | 44 |
| Prostate | 7 | 100 | 0 | 0 |
| Skin | 1 | 100 | 0 | 0 |
| Stomach | 8 | 89 | 1 | 11 |
| Tonsil^e^ | 0 | 0 | 10 | 100 |
| CNS^f^ | 9 | 100 | 0 | 0 |
| Liver | 9 | 100 | 0 | 0 |

^a^ Bronchial lumnial cells positive. ^b^ Inflammatory cells, endothelium and alevolar macrophages positive. Alveolar epithelium negative. ^c^ Positive staining in some lymphocytes & histiocytes. ^d^ Acinar pancreas positive staining. Ducts and langerhans islets negative. ^e^ Lymphoid cells (predominantly T cells), other inflammatory cells & endothelium positive. ^f^ Endothelial cells positive.

*Supplementary Table 3: Characteristics of the CRC. 0% cut-off used to determine SLFN11 status.*

|  | CRC whole cohort | | | |
| --- | --- | --- | --- | --- |
|  | SLFN11 negative | | SLFN11 positive | |
|  | n | % | n | % |
| Total patients | 115 | 80 | 29 | 20 |
| Male | 59 | 51 | 19 | 66 |
| Female | 56 | 49 | 10 | 34 |
| Age: |  |  |  |  |
| 34-54 | 16 | 14 | 7 | 24 |
| 55-74 | 74 | 64 | 16 | 55 |
| 75+ | 25 | 22 | 6 | 21 |
| Disease Stage^a^: |  |  |  |  |
| Stage I | 0 | 0 | 0 | 0 |
| Stage II | 16 | 14 | 3 | 11 |
| Stage III | 18 | 16 | 2 | 7 |
| Stage IV | 71 | 70 | 18 | 62 |
| Grade: |  |  |  |  |
| 1 | 0 | 0 | 0 | 0 |
| 2 | 97 | 84 | 18 | 62 |
| 3 | 18 | 16 | 11 | 38 |
| Metastatic at surgery?^b^ |  |  |  |  |
| Yes | 65 | 57 | 17 | 59 |
| No | 37 | 43 | 6 | 41 |

^a^ Disease stage known for 128 patients. ^b^ Metastatis at surgery known for 125 patients

*Supplementary Table 4: Characteristics of the 124 SCLC samples. 122 H-score cut-off used to determine SLFN11 status.*

|  | SCLC whole cohort | | | |
| --- | --- | --- | --- | --- |
|  | SLFN11 low | | SLFN11 high | |
|  | n | % | n | % |
| Total patients | 43 |  | 81 |  |
| SLFN11 sub-clonal | 7 | 16 | 12 | 15 |
| Male | 26 | 60 | 52 | 64 |
| Female | 17 | 40 | 27 | 33 |
| Smoker | 23 | 53 | 47 | 58 |
| Non-smoker | 0 | 0 | 2 | 2 |
| Smoking status unknown | 20 | 47 | 32 | 40 |
| Age: |  |  |  |  |
| 35-54 | 2 | 5 | 5 | 6 |
| 55-74 | 34 | 79 | 63 | 78 |
| 75+ | 5 | 12 | 11 | 14 |
| Treatment history: |  |  |  |  |
| Chemotherapy (agent known)^a^ | 16 | 37 | 36 | 44 |
| Chemotherapy (agent unknown)^b^ | 7 | 16 | 9 | 11 |
| Radiotherapy only | 1 | 2 | 4 | 5 |
| Surgical intervention only | 9 | 21 | 9 | 11 |
| Unknown | 10 | 23 | 23 | 28 |
| Disease Stage^c^: |  |  |  |  |
| Stage I | 4 | 9 | 15 | 19 |
| Stage II | 7 | 16 | 6 | 7 |
| Stage III | 11 | 26 | 18 | 22 |
| Stage IV | 5 | 12 | 6 | 7 |

^a^ Chemotherapy includes cisplatin, cisplatin+etoposide, carboplatin+etoposide, borteozmib, cisplatin+irinotecan, topotecan, etoposide, adriamycin + cyclophophamide + etoposide. ^b^ Patients had chemotherapy, but the type was unknown. ^c^ Disease stage known for 72 patients.

*Supplementary Table 5: Characteristics of the 24 SCLC patients treated with first line platinum + etoposide, where follow-up survival data was available. 122 H-score cut-off used to determine SLFN11 status.*

|  | Patients receiving first line platinum+etoposide | | | |
| --- | --- | --- | --- | --- |
|  | SLFN11 low | | SLFN11 high | |
|  | n | % | n | % |
| Total patients | 8 |  | 16 |  |
| SLFN11 sub-clonal | 3 | 38 | 2 | 13 |
| Treatment history: |  |  |  |  |
| Cisplatin+etoposide | 5 | 63 | 6 | 38 |
| Carboplatin+etoposide | 3 | 38 | 10 | 63 |
| Disease Stage ^a^: |  |  |  |  |
| Stage I | 1 | 13 | 3 | 19 |
| Stage II | 0 | 0 | 2 | 13 |
| Stage III | 1 | 13 | 1 | 6 |
| Stage IV | 1 | 13 | 0 | 0 |

^a^ Disease stage known for only 9 patients.

*Supplementary Table 6: Characteristics of the 45 SCLC patients treated with any chemotherapy, where follow-up progression free survival (PFS) data was available. 122 H-score cut-off used to determine SLFN11 status.*

|  | Patients receiving chemotherapy* (with PFS data) | | | |
| --- | --- | --- | --- | --- |
|  | SLFN11 low | | SLFN11 high | |
|  | n | % | n | % |
| Total patients | 15 |  | 30 |  |
| SLFN11 sub-clonal | 3 | 20 | 5 | 17 |
| Disease Stage ^a^: |  |  |  |  |
| Stage I | 1 | 7 | 5 | 17 |
| Stage II | 1 | 7 | 3 | 10 |
| Stage III | 4 | 27 | 3 | 10 |
| Stage IV | 2 | 13 | 0 | 0 |

^a^ Disease stage known for 19 patients.

* Chemotherapy includes cisplatin, cisplatin+etoposide, carboplatin+etoposide, borteozmib, cisplatin+irinotecan, topotecan, etoposide, adriamycin + cyclophophamide + etoposide or patients had chemotherapy, but the type was unknown.

*Supplementary Table 7: Characteristics of the 57 SCLC patients treated with any chemotherapy, where follow-up overall survival (OS) data was available. 122 H-score cut-off used to determine SLFN11 status.*

|  | Patients receiving chemotherapy* (with OS data) | | | |
| --- | --- | --- | --- | --- |
|  | SLFN11 low | | SLFN11 high | |
|  | n | % | n | % |
| Total patients | 19 |  | 38 |  |
| SLFN11 sub-clonal | 4 | 21 | 5 | 13 |
| Disease Stage ^a^: |  |  |  |  |
| Stage I | 1 | 5 | 5 | 13 |
| Stage II | 1 | 5 | 3 | 8 |
| Stage III | 6 | 32 | 9 | 24 |
| Stage IV | 2 | 11 | 1 | 3 |

^a^ Disease stage known for 28 patients.

* Chemotherapy includes cisplatin, cisplatin+etoposide, carboplatin+etoposide, borteozmib, cisplatin+irinotecan, topotecan, etoposide, adriamycin + cyclophophamide + etoposide or patients had chemotherapy, but the type was unknown.

*Supplementary Table 8: BRCA and HRR mutation status of the 110 HGSOC patients treated with either olaparib or placebo in study 19, where tissue was evaluable for SLFN11 by IHC. A H-score cut-off of 30 was used to determine SLFN11 status.*

|  | SLFN11 high (≥31 H-score) | | | | SLFN11 low (<31 H-score) | | | |
| --- | --- | --- | --- | --- | --- | --- | --- | --- |
|  | Olaparib | | Placebo | | Olaparib | | Placebo | |
|  | n | % | n | % | n | % | n | % |
| Females | 21 | 100 | 18 | 100 | 34 | 100 | 37 | 100 |
| **BRCAm** | 12 | 57 | 9 | 50 | 13 | 38 | 21 | 57 |
| **BRCAwt** | 8 | 38 | 9 | 50 | 11 | 32 | 15 | 41 |
| *HRRm* | *5* | *24* | *3* | *17* | *4* | *12* | *2* | *5* |
| *HRRwt* | *2* | *10* | *5* | *28* | *10* | *29* | *11* | *30* |
| *Unknown* | *1* | *5* | *1* | *6* | *6* | *18* | *2* | *5* |
| **BRCAm+HRRm** | 17 | 81 | 12 | 67 | 17 | 50 | 23 | 62 |
| **Undertermined** | 1 | 5 | 0 | 0 | 1 | 3 | 1 | 3 |

BRCA status undetermined for n=3 patients.


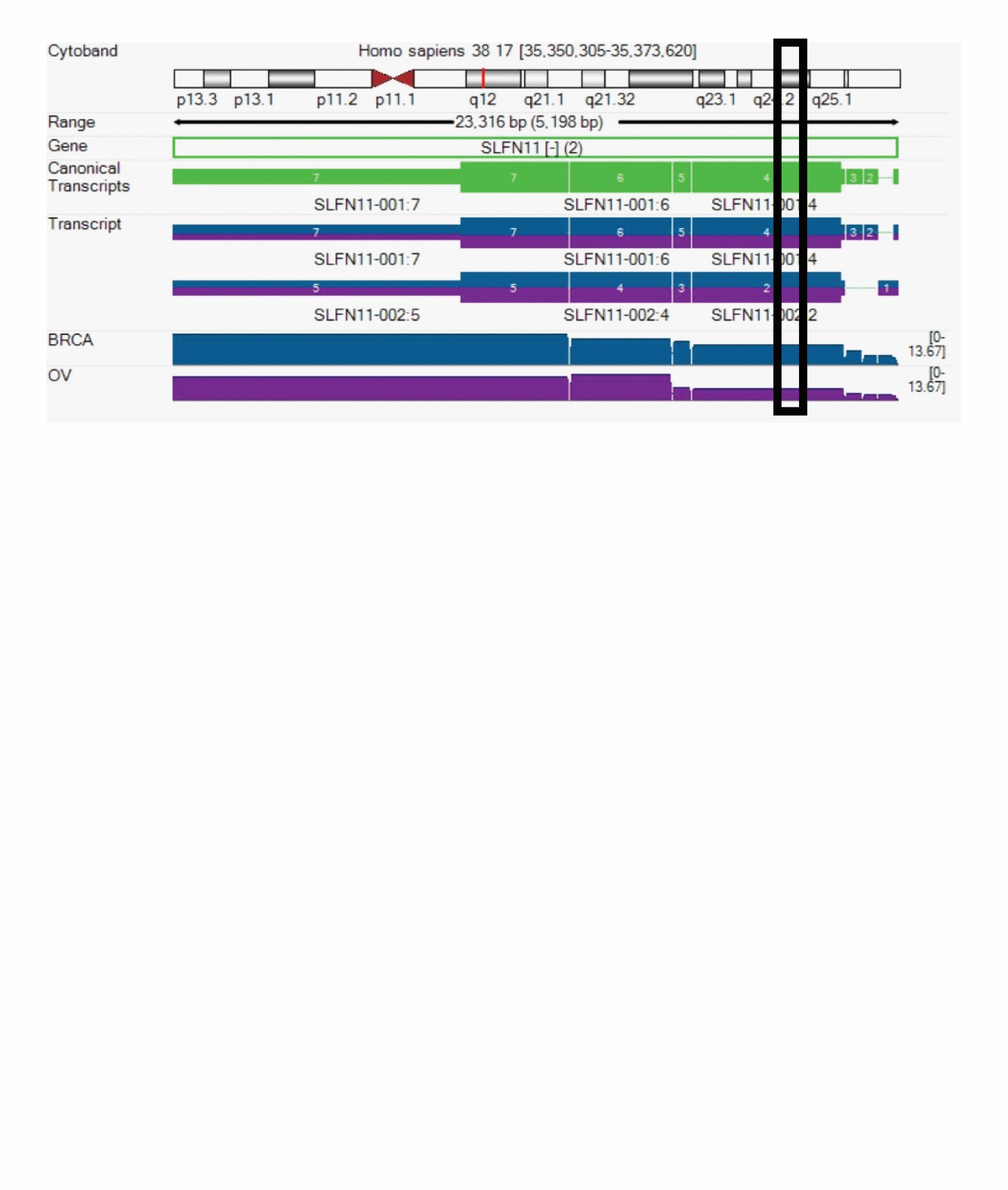


*Supplementary Figure 1. SLFN11 NanoString Probe design*

OmicSoft Array Studio Genome Browser was used to visualize gene expression data. SLFN11 NanoString probe location is highlighted by black box, this region is expressed in both breast and ovarian cancer patients.


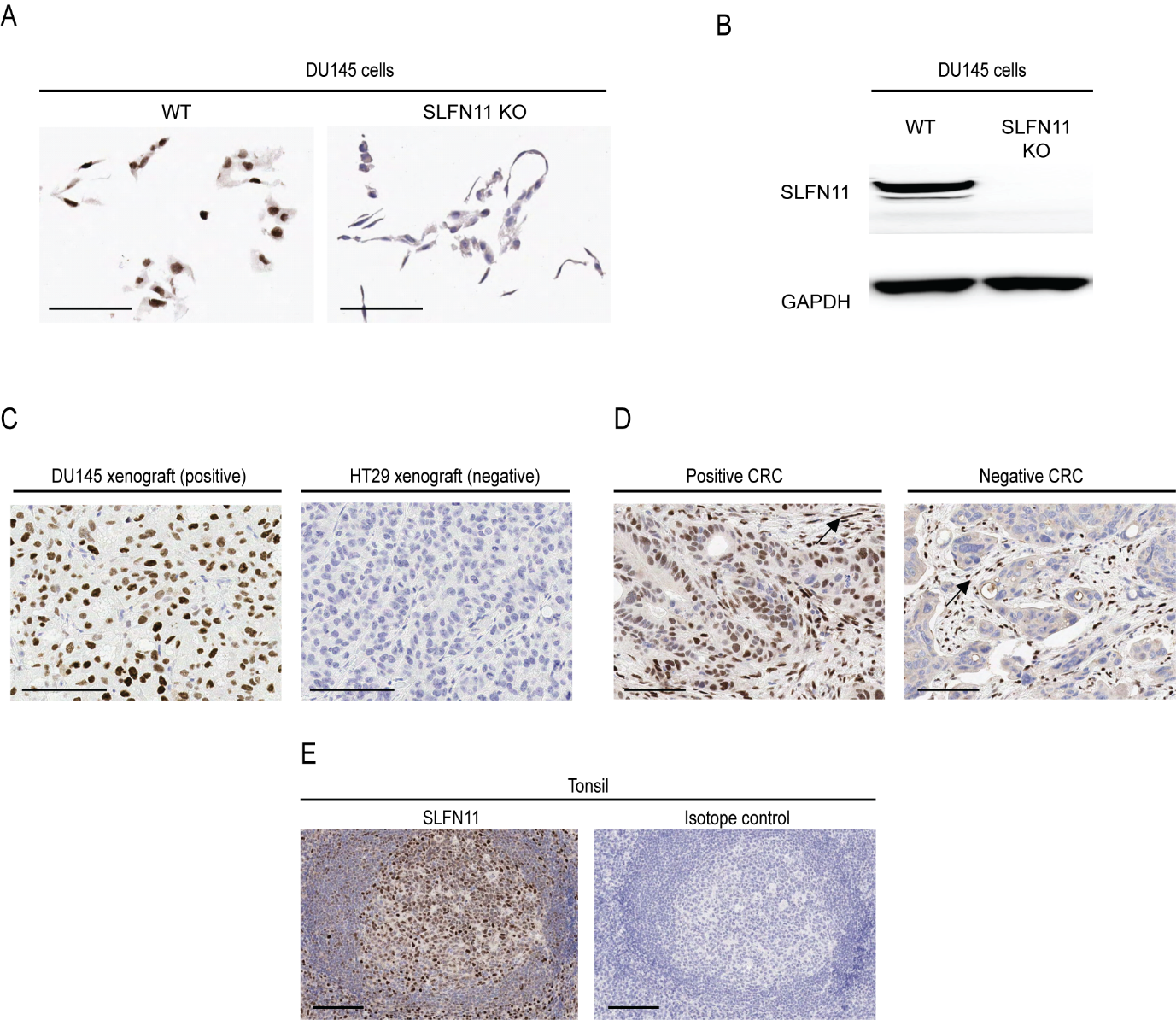


*Supplementary Figure 2: SLFN11 can be specifically and reliably detected in clinical tissues by IHC, using stromal and endothelial cells as internal tissue controls.*

SLFN11 protein expression in DU145 WT and CRISPR-Cas9 SLFN11 knock-out (KO) cells by IHC (**A**) and Western blot (**B**), demonstrate the specificity of the antibody. (**C**) SLFN11 positive prostate cancer DU145 xenograft and negative colorectal cancer HT29 xenograft showing expected SLFN11 nuclear staining pattern. (**D**) SLFN11 positive and negative colorectal cancer (CRC) human tissues surrounded by positive internal tissue control stromal and endothelial cells. (**E**) IHC staining of a SLFN11 positive tonsil control compared to a serial section stained with a rabbit IgG isotype control. Black arrows are stromal and endothelial cells. Scale bars at 100 µm.


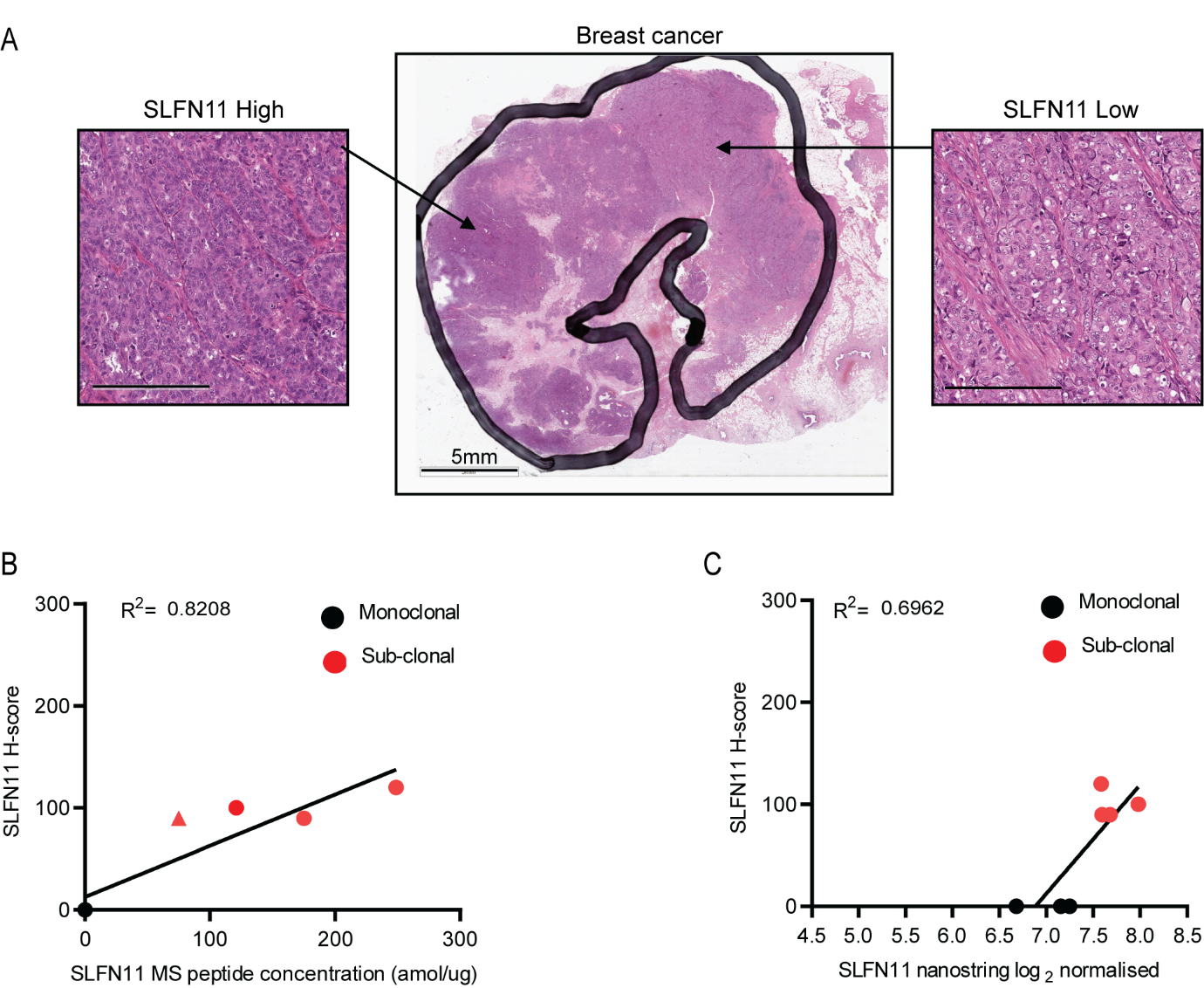


*Supplementary Figure 3: Broad comparability between protein and gene expression detection methods of SLFN11 in breast cancer and ovarian cancer human tissues.*

(**A**) Hematoxylin and Eosin (H&E) stain of a SLFN11 sub-clonal breast tumour, with the SLFN11 positive and negative sub-clones magnified, and tumour area indicated within the mark-up. Scale bars at 200 µm, unless otherwise indicated. (**B**) The immunohistochemistry (IHC) H-score and mass spectrometry (MS) protein quantification of SLFN11 expression present in each breast cancer tissue. (**C**) IHC H-score and gene expression by NanoString comparison in breast cancer tissue. Red point shows sub-clonal expression. Black point shows monoclonal expression. LLOQ is estimated to have a peptide concentration of 75 amol/µg (between LOQ and LLOQ), shown by a triangle. Linear regression line and R^2^ shown on the graphs.

*
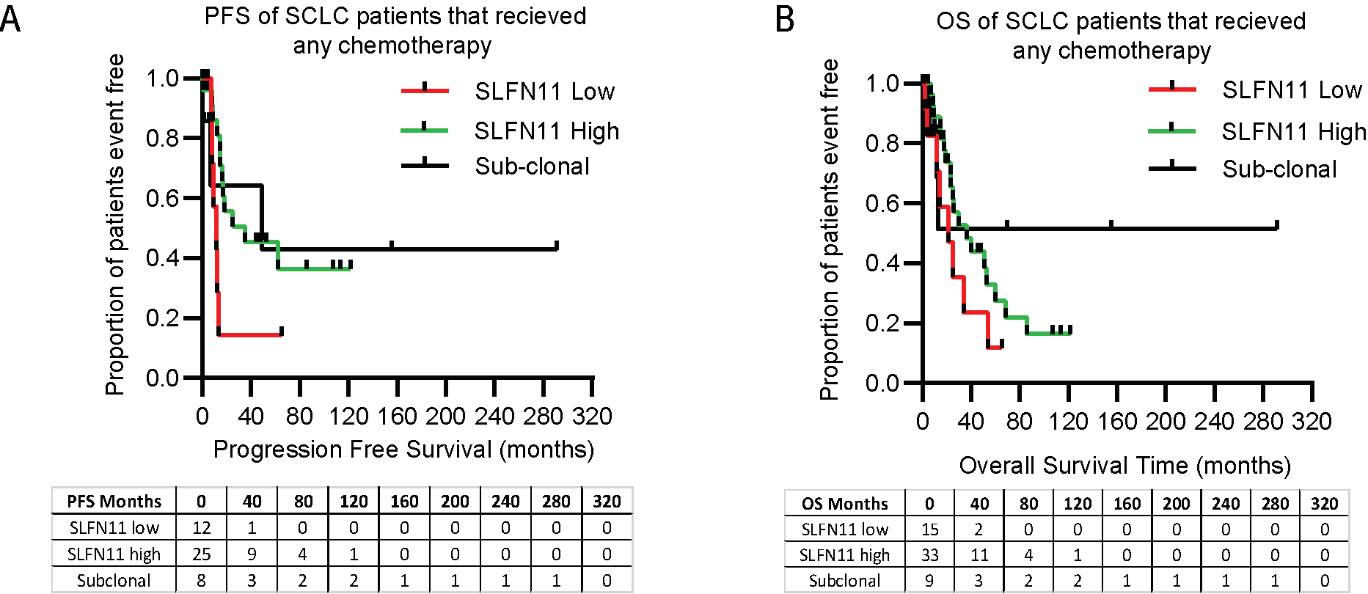
*

*Supplementary Figure 4: SLFN11 sub-clonality not associated with clinical outcome in SCLC.*

(**A-B**) Kaplan-Meier analysis of SCLC patients categorised by SLFN11 expression; SLFN11 high (H-score >122) compared to low (H-score ≤122) compared to SCLC patients with sub-clonal SLFN11 expression. (**A**) Progression free survival (PFS) and (**B**) overall survival (OS) in months for patients that received any chemotherapy. Events table with patients at risk shown for each timepoint.


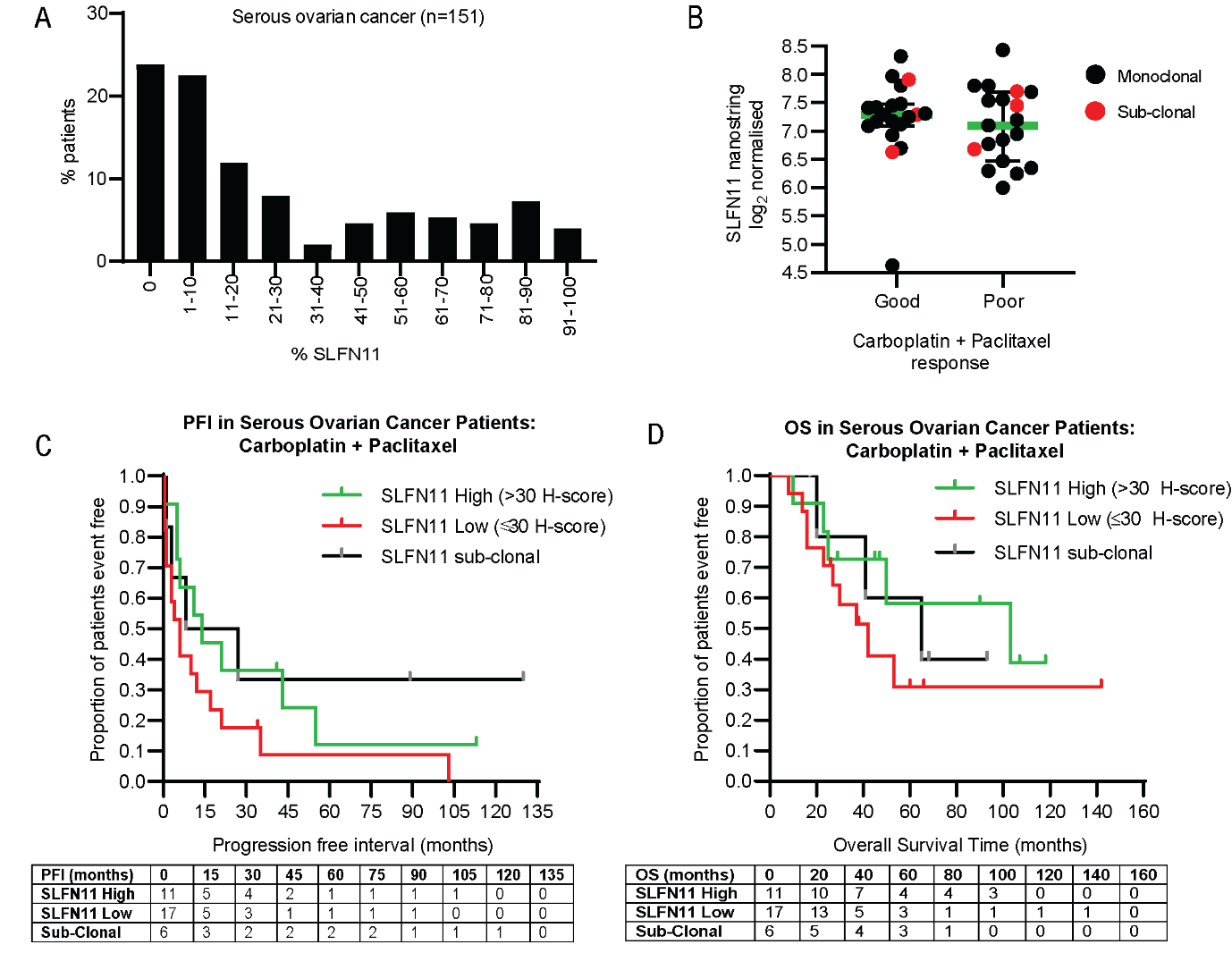


*Supplementary Figure 5: SLFN11 and SLFN11 sub-clonality not significantly associated with sensitivity to the paclitaxel-carboplatin doublet in HGSOC.*

(**A**) Histogram demonstrating distribution of SLFN11 expression by IHC positivity across n=151 serous ovarian cancer. (**B**) SLFN11 expression by NanoString gene expression in extreme HGSOC clinical responders to carboplatin and paclitaxel doublet therapy. Median ± interquartile range shown. (**C**) PFI Kaplan-Meier using 30 H-score cut-off for SLFN11 and a separate group for patients demonstrating sub-clonal SLFN11 expression. (**D**) Overall Survival (OS) of high vs. low vs. sub-clonal SLFN11 groups. Events table with patients at risk shown for each timepoint.


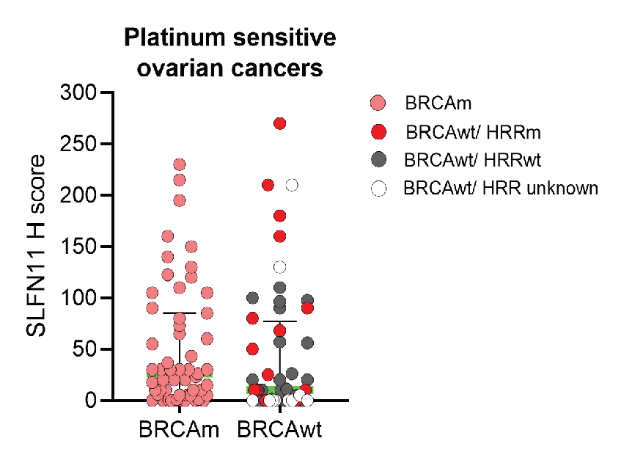


*Supplementary Figure 6. SLFN11 cut-off critical in understanding if SLFN11 confers sensitivity to olaparib.*

SLFN11 expression by H-score in the platinum sensitive serous ovarian cancers subcategorised with BRCA and homologous recombination repair (HRR) status (m; mutant vs. wt; wild type). Patients that were BRCAwt, but HRR was unknown are also included. Median ± interquartile range shown.
